# Aerobic exercise training improves not only brachial artery flow‐mediated vasodilatation but also carotid artery reactivity: A randomized controlled, cross‐over trial in older men

**DOI:** 10.14814/phy2.15395

**Published:** 2022-08-27

**Authors:** Jordi P. D. Kleinloog, Ronald P. Mensink, Jos op’t. Roodt, Dick H. J. Thijssen, Matthijs K. C. Hesselink, Peter J. Joris

**Affiliations:** ^1^ Department of Nutrition and Movement Sciences, NUTRIM School of Nutrition and Translational Research in Metabolism Maastricht University Maastricht The Netherlands; ^2^ Department of Internal Medicine Maastricht University Medical Centre Maastricht The Netherlands; ^3^ Department of Physiology Radboud University Medical Centre Nijmegen The Netherlands; ^4^ Research Institute for Sport and Exercise Sciences Liverpool John Moores University Liverpool UK

**Keywords:** aerobic exercise, aging, arterial stiffness, endothelial function

## Abstract

It is well‐known that aerobic exercise training beneficially affects endothelial function as measured by brachial artery flow‐mediated vasodilation (FMD). This trial with older sedentary overweight and obese men, therefore, examined the effects of aerobic training on other non‐invasive markers of the vasculature, which have been studied in less detail. Seventeen men (67 ± 2 years, BMI: 30.3 ± 2.8 kg/m^2^) participated in this controlled cross‐over study. Study participants followed in random order a fully supervised, progressive, aerobic exercise training (three 50‐min sessions each week at 70% maximal power) and a no‐exercise control period for 8 weeks, separated by a 12‐week wash‐out period. At the end of each period, endothelial function was assessed by the carotid artery reactivity (CAR) response to a cold pressor test and FMD, and local carotid and regional aortic stiffness by the carotid‐to‐femoral pulse wave velocity (PWV_c–f_). The retinal microvasculature, the serum lipid profile, 24‐h ambulatory blood pressure, and 96‐h continuous glucose concentrations were also determined. Aerobic training increased CAR from 1.78% to 4.01% (Δ2.23 percentage point [pp]; 95% CI: 0.58, 3.89 pp; *p* = 0.012) and FMD from 3.88% to 6.87% (Δ2.99 pp; 95% CI: 0.58, 5.41 pp; *p* = 0.019). The stiffness index β_0_ increased by 1.1 (95% CI: 0.3, 1.9; *p* = 0.012), while PWV_c–f_ did not change. Retinal arteriolar width increased by 4 μm (95% CI: 0, 7 μm; *p* = 0.041). Office blood pressure decreased, but ambulatory blood pressure, and serum lipid and continuous glucose concentrations did not change. Aerobic exercise training improved endothelial function and retinal arteriolar width in older sedentary overweight and obese men, which may reduce cardiovascular risk.

## Graphical abstract

Through effects on vascular markers, age‐related health problems can be prevented. Aerobic exercise improves vascular function and retinal structure in older men. Training improves endothelial function in an elastic conduit and muscular artery. Blood pressures improves, but lipid profile was not affected. Exercise‐induced changes in vasculature may reduce cardiovascular disease risk.

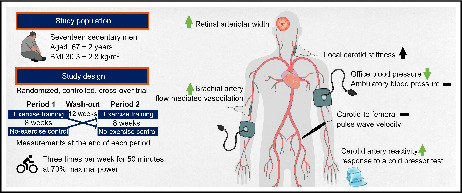

## 1 INTRODUCTION

Physical exercise training is a well‐known strategy to prevent age‐related health problems, such as cardiovascular disease (CVD) and cognitive decline (Benjamin et al., 2018; Rapsomaniki et al., 2014). A common denominator of these comorbidities is an impaired vascular function (Gorelick et al., 2011), which can be measured with different non‐invasive techniques, each addressing a different aspect of the vasculature (Benjamin et al., 2018; Cohn et al., 2004). Most well‐controlled trials investigating the effects of exercise training on vascular function have however focused on only one specific characteristic of the vasculature (Seals et al., 2019). Thus, it has frequently been shown that vascular endothelial function of a peripheral muscular artery as measured by shear stress‐induced brachial artery flow‐mediated vasodilation (FMD) is improved after exercise training (Early et al., 2017; Pedralli et al., 2020; Qiu et al., 2018; Son et al., 2017). However, the endothelial function can also be examined in a major elastic conduit artery by assessing carotid artery reactivity (CAR), which involves stimulation of the sympathetic nervous system using a cold pressor test (van Mil et al., 2017). CAR correlates with coronary artery responses to a cold pressor test, an independent predictor of cardiovascular events (van Mil et al., 2017; Van Mil et al., 2019). The effects of exercise training on CAR are not known.

In addition, only a limited number of studies have examined the effects of exercise on parameters reflecting local carotid stiffness, but the results are inconsistent (Tanaka, 2019). Regional aortic arterial stiffness, as determined by the current non‐invasive gold standard method of carotid‐to‐femoral pulse wave velocity (PWV_c–f_), is a well‐established risk marker of CVD (Sutton‐Tyrrell et al., 2005). However, more pronounced reductions in brachial‐ankle as compared with carotid‐femoral pulse wave velocity (PWV) responses were observed after aerobic exercise training (Ashor et al., 2014). Further, exercise‐induced beneficial effects on the retinal microvasculature, imaged using fundus photography, are related to a reduced cardiovascular risk in obese adults (Hanssen et al., 2011). Finally, macrovascular complications (i.e., early signs of atherosclerotic plaque formation) were evaluated by the assessment of carotid intima‐media thickness (cIMT) (Touboul et al., 2012). Exercise training may have differential effects on different aspects of the vascular tree (e.g., vascular endothelial function, arterial stiffness, and structure) because different underlying mechanisms may be involved. Therefore, applying all these non‐invasive techniques provides a more complete picture on the true effects of exercise training on the vasculature.

Recently, we have already reported that in sedentary overweight and obese older men a fully controlled aerobic exercise training protocol improved regional cerebral blood flow (CBF) (Kleinloog et al., 2019), which reflects cerebrovascular function. We here report aerobic‐training effects on the central (i.e., carotid artery and aorta), peripheral (i.e., brachial artery), and retinal microvasculature that were assessed using different non‐invasive markers for endothelial function, arterial stiffness, and vascular structure. Traditional cardiometabolic risk markers were also determined and used to calculate the Framingham Risk score as a predictor for general cardiovascular risk (D'Agostino Sr. et al., 2008). Finally, continuous measurements during daily life of physical activity and blood pressure levels, and glucose concentrations were measured. This integrated approach will further contribute to the understanding of the beneficial effects of exercise on CVD risk, which cannot only be explained by the traditional CVD risk markers (Green et al., 2008).

## 2 METHODS

### 2.1 Study participants and design

Sedentary older overweight and obese men participated in a randomized, controlled cross‐over trial with an aerobic exercise intervention and a no‐exercise control period of both 8 weeks, separated by a 12‐week wash‐out period. An overview of the study design is shown in Figure S1. Participants were allocated based on a computer‐generated randomization scheme. Participants and investigators were unaware of the allocation prior to inclusion but could not be blinded during the intervention and measurements. However, images and blood samples were blinded prior to analysis. Study details have been described before (Kleinloog et al., 2019). In brief, men were included if they met the following criteria: aged between 60 and 70 years, body mass index (BMI) between 25 and 35 kg/m^2^, no chronic diseases, no use of medication affecting the outcome measures, systolic (SBP) < 160 mmHg and diastolic blood pressure (DBP) < 100 mmHg, and a low physical activity level using the long form of the Dutch version of the international physical activity questionnaire (IPAQ; Craig et al., 2003; IPAQ Research Committee, 2005). This version (31 items) collects detailed information on the duration and frequency of physical activity levels within five different domains (job‐related, transport‐related, housework‐related, leisure‐time, and sedentary behavior). The intervention period consisted of a fully supervised, personalized, and progressive aerobic‐based exercise program on a cycling ergometer three times a week for 50 min. The training comprised 10 min warm‐up at 45% maximal workload (Pmax), 30 min at 70% Pmax, and 10 min cool‐down at 45% Pmax. Maximal exercise capacity was determined during incremental cycling before the start of the training intervention period, at weeks 2, 4, and 6, and the end of the training intervention period. During these tests, peak oxygen consumption (VO_2_peak) was measured as well, which was used to adjust training intensity for the next 2 weeks. During the control and wash‐out periods, participants had to maintain or return to their habitual physical activity levels. Men were requested not to change their habitual diet and consumption of alcohol throughout the study period, which was checked using a food frequency questionnaire. Energy and nutrient intakes were calculated using the Dutch Food Composition table.

Outcome variables were measured at the start of the control and intervention periods (BL), after 4 weeks (WK4), and during a follow‐up day (FU‐1) at the end of both periods. Vascular function and blood pressure were assessed 43 h (range: 19–72 h) after the final training. Additional blood samples were taken 117 h (range: 70–118 h) after FU‐1 during a second follow‐up day (FU‐2). Participants arrived after an overnight fast and were requested to have a regular meal the evening before, and to refrain from alcohol and exercise 24 h prior to each visit. Between both follow‐up days, ambulatory blood pressure (ABP) levels were monitored, continuous glucose measurement (CGM) was performed, and physical activity was measured using accelerometry.

The study followed the ethical guidelines of the Declaration of Helsinki and was approved by the Medical Ethics Committee of Maastricht University Medical Centre (METC‐173025). All study participants gave written informed consent before the start of the intervention trial. This study was registered at ClinicalTrials.gov (registry number: NCT03272061) on September 7, 2017.

### 2.2 Anthropometrics

Height was measured during the screening visit using a wall‐mounted stadiometer (Seca 222; Seca) and scale (Seca 877; Seca). Body weight was measured every 2 weeks in the intervention period and at BL, WK4, and FU‐1 in the control period. BMI was calculated and body fat distribution was assessed by measuring the waist‐to‐hip circumference ratio (Seca 201; Seca).

### 2.3 Vascular measurements

Vascular function measurements were performed at FU‐1 after a resting period of at least 15 min in the supine position in a temperature‐controlled, quiet, and darkened room at the Metabolic Research Unit Maastricht (MRUM).

#### 2.3.1 Central vasculature

Ultrasound imaging in B‐mode using a 13‐MHz transducer (MyLab Gamma; Esaote) with continuous recording was used to visualize the left common carotid artery 2 cm proximal to the bifurcation of the common carotid artery into the internal and external carotid arteries. The CAR in response to a cold pressor test was determined. The cold pressor test consisted of a 1‐min baseline period and 3‐min immersion of the hand in a bucket of cold water (4.0°C) with ice slush. The carotid artery baseline diameter was defined as the average diameter over the first minute. Diameters were averaged over 10‐s intervals during immersion (Buckley et al., 2019). The maximal percentage change in post‐immersion arterial diameter relative to the baseline arterial diameter was calculated. Additionally, the change in diameter was allometrically scaled and corrected for the baseline diameter as previously described (Atkinson & Batterham, 2013). The change relative to baseline was also determined for every interval of 10 s to calculate the net incremental area under the curve (net iAUC). The echo images were analyzed offline with a custom‐written MATLAB program using automated edge‐detection and wall tracking (MyFMD V15.06, AP Hoeks, Department of Biomedical Engineering, Maastricht University Medical Centre, Maastricht, Netherlands). During the baseline period, five‐to‐six heartbeats were analyzed to determine the cIMT, and systolic and diastolic diameters of the carotid artery using a custom‐written MATLAB program (VidArt V13.5, AP Hoeks, Department of Biomedical Engineering, Maastricht University Medical Centre, Maastricht, Netherlands). Local arterial stiffness was determined using pressure‐independent stiffness index β_0_ (Spronck et al., 2017). The used formula is: $\beta_{0}=\frac{\ln\left({\mathrm{SBP}}_{c}/{\mathrm{DBP}}_{c}\right)}{\left(D_{s}/D_{d}\right)-1}-\ln\left(\frac{{\mathrm{DBP}}_{c}}{{\mathrm{BP}}_{\mathrm{ref}}}\right)$. Where *D*_s_ and *D*_d_ are the carotid arterial systolic and diastolic diameter and cIMT is the intima‐media thickness of the carotid artery. The average DBP for both periods from all participants was used as reference blood pressure (BP_ref_).

PWV_c–f_ was determined in triplicate with a tonometer (SphygmoCor v9; AtCor Medical) according to the current guidelines (Townsend, 2017). The direct distance between the left carotid and femoral artery was used. Additionally, radial artery pulse wave analyses (PWA) were performed near the wrist of the arm in triplicate using the same tonometer. Central augmentation index corrected for heart rate (CAIxHR75) was determined as described (Townsend, 2017).

#### 2.3.2 Peripheral vasculature

FMD was also assessed by ultrasound echography in B‐mode using a 13‐MHz transducer (MyLab Gamma; Esaote) with continuous recording as recommended (Thijssen et al., 2019). After a resting baseline period of 3 min, a pneumatic cuff placed around the forearm of the participant was inflated to 200 mmHg for 5 min. Response of the brachial artery diameter following reactive hyperemia was imaged for another 5 min. FMD was quantified as the maximal percentage change in post‐occlusion arterial diameter relative to baseline diameter. Additionally, allometric scaling was performed to correct for differences in baseline diameters expressed as equivalent FMD percentage (Atkinson & Batterham, 2013). The B‐mode images were analyzed offline with the same software as for the CAR.

#### 2.3.3 Retinal microvasculature

Retinal vascular images were made to assess microvascular calibers in the eye as described previously (Joris et al., 2017). The nonmydriatic retinal camera (Topcon TRC‐NW‐300; Topcon Co.) focused on the right optic disc and photographed the retina. Images were digitally analyzed to calculate mean central retinal arteriolar (CRAE) and venular equivalents (CRVE), and the arteriolar‐to‐venular ratio (AVR) using the Parr‐Hubbard formulas (Hubbard et al., 1999) and appropriate software (Generalized Dual‐Bootstrap Iterative Closest Point [Stewart et al., 2003]). Retinal images from the intervention and control periods were analyzed simultaneously to ensure that the same segments from at least two arteries and venules were used for a participant.

### 2.4 Cardiometabolic risk markers

Office brachial SBP and DBP were measured four times using an intermittent blood pressure device (Omron Intellisense M7; Cemex Medische Techniek). The mean of the last three measurements is reported. The mean arterial pressure (MAP) was determined using the pulse wave that was determined at the brachial artery near the antecubital fossa with a tonometer (SphygmoCor v9; AtCor Medical). Additionally, central systolic and diastolic blood pressure (SBP_c_ and DBP_c_) were determined using the radial pulse wave based on the brachial DBP and MAP. ABP was also measured (Mobil‐O‐Graph; I.E.M. Inc.). Brachial blood pressure levels were recorded every 15 min during the daytime (07:00 h till 22:00 h) and every 30 min at night (22:00 h till 07:00 h). The first measurement was discarded, and the mean and variability (i.e., SD) of the SBP, DBP, pulse pressure (PP), and heart rate (HR) were calculated over 24‐h, and during the daytime and nighttime. Additionally, nocturnal SBP and nocturnal DBP dipping were calculated using the mean difference between daytime and nighttime blood pressure expressed as a percentage of the daytime value.

Fasting blood samples were taken from a forearm vein at BL, WK4, and FU‐1. At FU‐2, blood samples were taken using an intravenous cannula. Blood drawn in vacutainer SST™ II Advance tubes (Becton, Dickson and Company) were allowed to clot for at least 30 min at 21°C. Vacutainer tubes were centrifuged at 1300 *g* for 15 min at 21°C to obtain serum. Tubes containing sodium fluoride (NaF) plus Na_2_EDTA (Becton, Dickson and Company) were kept on ice and centrifuged for 30 min at 1300 *g* for 15 min at 4°C to obtain plasma. Serum and plasma samples were immediately portioned, frozen in liquid nitrogen, and stored at −80°C until analysis at the end of the study. Serum samples were analyzed for concentrations of total cholesterol (TCH: CHOD‐PAP method; Roche Diagnostics), triacylglycerol corrected for free glycerol (TAG: GPO Trinder; Sigma‐Aldrich Corporation), HDL‐cholesterol (precipitation method; Roche Diagnostics), and high‐sensitivity C‐reactive protein (hsCRP) (immunoturbidimetric assay, Horiba ABX, Montpellier). LDL‐cholesterol was calculated using the Friedewald formula. Values of FU‐1 and FU‐2 were averaged before statistical analyses. Additionally, the Framingham Risk score was calculated using the values at the end of the intervention and control periods (D'Agostino Sr. et al., 2008).

### 2.5 Continuous glucose monitoring and physical activity

Glucose concentrations were measured every 15 min using the Freestyle Libre Pro (Abbott), which was placed precisely at the back of the upper arm. The AUC and iAUC were calculated with the trapezoid rule over a 96‐h period using GraphPad Prism 8 (FAO/WHO, 1998). The baseline glucose concentration for the iAUC was determined by taking the average minimal 1‐h value of each measurement day.

Accelerometry‐based physical activity levels were measured using the activPAL3 (PAL Technologies Ltd), a validated accelerometer for measuring physical activity and sedentary behavior (Edwardson et al., 2017). The accelerometer was placed on the anterior side of the left thigh 10 cm above the patella to monitor daily activity patterns and was worn uninterrupted for a median duration of 120 h (range: 95–144 h). Data were processed using the PAL analysis software with the CREA algorithm (Version 8.11.4.61; PAL Technologies Ltd). Physical activity score expressed as the metabolic equivalent time of task per second (MET/s) and total sedentary time in seconds was extracted from the 15‐s epoch file.

### 2.6 Statistical analyses

Differences at baseline were investigated using repeated analysis of variance (ANOVA) with a period as a fixed factor. When measurements were only performed at the end of the intervention and control periods, the effects of the exercise program were also examined using repeated ANOVA with a period as a fixed factor. Linear mixed models were performed for serum lipids and hsCRP to test for differences between treatments over time, using absolute changes from baseline as a dependent variable. Time, treatment, period, and time × treatment interaction were used as fixed factors, and participant and intercept as random factors. The interaction term was omitted from the model if it was not significant. When the time × treatment interaction was statistically significant, the difference in changes at weeks 4 and 8 between the intervention and control periods were compared pairwise with Bonferroni correction. Unless otherwise indicated, normally distributed variables are shown as means (±SDs), and non‐normal distributed variables are shown as medians (ranges). Concentrations of hsCRP were log‐transformed, as concentrations were not normally distributed based on quantile‐quantile plots. Pearson correlations were determined between changes in peripheral and changes in central artery function, and between the change in blood pressure and changes in the retinal microvasculature. Only variables that changed significantly were correlated. SPSS was used to perform all statistical analyses (IBM Corp., IBM SPSS Statistics, V26). A *p* < 0.05 was considered to be statistically significant.

## 3 RESULTS

### 3.1 Study participants

A consolidated standard of reporting trials (CONSORT) flow diagram of study participants is shown in Figure S2. A total of 17 participants completed the study. FMD measurements for two participants and one CAR measurement could not be analyzed due to technical problems with the recording of the images. Another FMD assessment could not be analyzed due to the insufficient quality of the ultrasound recordings. Accelerometer recordings of one participant were missing because the device was lost during the recordings. Finally, the CGM measurements of one study participant failed.

Baseline characteristics of the study participants, who completed the study have been described before (Kleinloog et al., 2019). The men had a mean age of 67 ± 2 years and their mean BMI was 30.3 ± 2.8 kg/m^2^. The median attendance rate of the scheduled training sessions was 100% (range: 92%–100%). Weight and waist‐to‐hip circumference ratio did not differ between treatments (treatment effects: *p* = 0.830 and *p* = 0.823, respectively) and remained stable throughout the study (time effects: *p* = 0.289 and *p* = 0.373, respectively). As anticipated, aerobic fitness improved, as indicated by a significant time × treatment interaction (*p* = 0.018) for the VO_2_peak, which tended to increase by 99 ml/min after 4 weeks (95% CI: −15, 214 ml/min; *p* = 0.088) and increased significantly by 262 ml/min after 8 weeks (95% CI: 153, 394 ml/min; *p* < 0.001) (Kleinloog et al., 2019). Energy and nutrient intakes did also not change during the study (Table S1).

### 3.2 Vascular measurements

#### 3.2.1 Central vasculature

**FIGURE 1:**
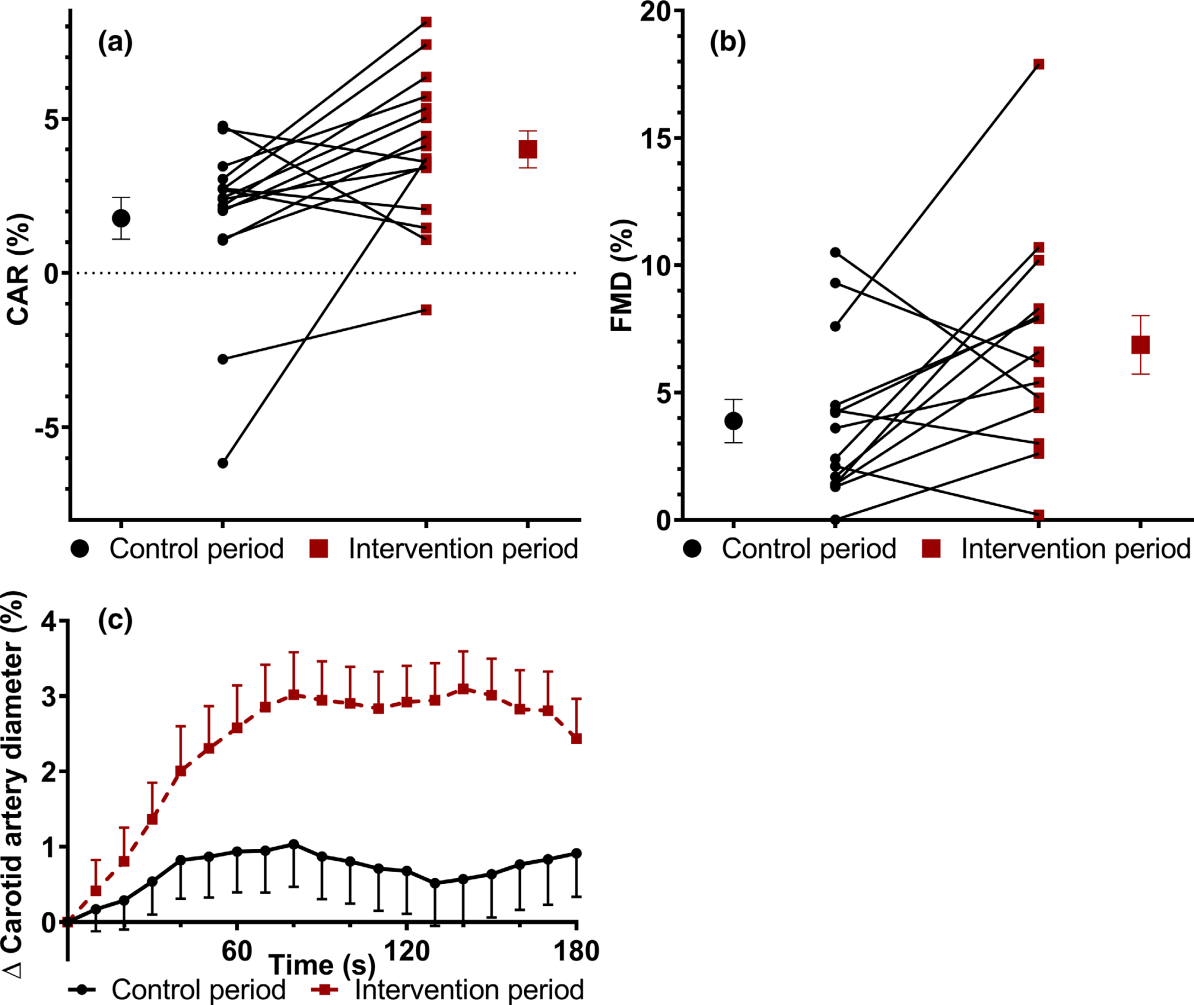
Training‐induced changes of endothelial function markers of a randomized, controlled cross‐over study with sedentary overweight and obese men. Mean (±SEM) and individual (a) carotid artery responses (CAR; *n* = 16) and (b) brachial artery flow‐mediated vasodilation (FMD; *n* = 14) following the control and intervention period. (c) Mean (±SEM) carotid artery diameter changes averaged for every 10 s during the cold pressure test (*n* = 16) following the control and intervention period.

CAR responses were 4.01% after exercise training and 1.78% after the control period and thus improved by 2.23 percentage points (pp) (95% CI: 0.58, 3.89 pp; *p* = 0.012). Allometrically scaled CAR responses increased by 2.68 pp (95% CI: 1.11, 4.19 pp; *p* = 0.003). Additionally, the net iAUC increased by 104%*min (95% CI: 35, 173 pp*min; *p* = 0.006). Baseline carotid artery diameters before the cold pressor test, but also diastolic and systolic diameters over five‐to‐six heart beats did not differ (Table 1; Figure 1a,c). Moreover, exercise training increased local arterial stiffness, as indicated by an increased stiffness index β_0_ (Δ 1.1; 95% CI: 0.3, 1.9; *p* = 0.010) (Table 1). As expected, cIMT (Δ 0.02 mm; 95% CI: −0.05, 0.08 mm; *p* = 0.579) and the lumen‐to‐cIMT ratio did not change (Δ 0.02; 95% CI: −0.27, 0.34; *p* = 0.816). In contrast, PWV_c–f_ (Δ 0.4 m/s; 95% CI: −0.4, 1.2 m/s; *p* = 0.264) and CAIxHR75 (Δ −0.8 pp; 95% CI: −2.9, 1.4 pp; *p* = 0.448) did not significantly change (see Table 1).

**TABLE 1:** Vascular markers, blood pressure, and continuous glucose measurement outcomes from a randomized, controlled cross‐over study with sedentary older men

	Intervention period	Control period	Mean difference (95% CI)	
**Vascular measurements**				
CAR (%)^a^	4.01 ± 2.41	1.78 ± 2.71	2.23	(0.58, 3.89)*
Corrected CAR (equivalent %)^a^	4.19 ± 2.39	1.51 ± 2.83	2.68	(1.11, 4.19)**
CAR_net iAUC_ (%*min)^a^	42 ± 116	147 ± 106	104	(35, 173)**
Carotid baseline diameter (mm)^a^	7.76 ± 1.43	7.71 ± 1.35	0.05	(−0.07, 0.18)
Brachial artery FMD (%)^b^	6.87 ± 4.32	3.88 ± 3.19	2.99	(0.58, 5.41)*
Corrected FMD (equivalent %)	6.40 ± 4.05	4.19 ± 3.07	2.21	(−1.69, 6.18)
Brachial baseline diameter (mm)^b^	3.25 ± 0.34	3.59 ± 0.52	−0.34	(−0.45, −0.17)**
Carotid diastolic diameter (mm)^a^	8.22 ± 1.02	8.08 ± 0.96	0.12	(0.00, 0.25)*
Carotid systolic diameter (mm)^a^	8.54 ± 1.11	8.52 ± 1.09	0.02	(−0.09, 0.14)
Stiffness index β_0_ ^a^	7.4 ± 1.6	6.1 ± 2.1	1.1	(0.3, 1.9)*
PWV_c–f_ (m/s)	12.6 ± 2	12.3 ± 1.8	0.4	(−0.4, 1.2)
CAIxHR75 (%)	19.5 ± 7.8	20.7 ± 7.4	−0.8	(−2.9, 1.4)
CRAE (μm)	119 ± 27	115 ± 27	4	(0, 7)*
CRVE (μm)	194 ± 32	193 ± 33	1	(−1, 3)
AVR ratio	0.61 ± 0.1	0.60 ± 0.11	0.02	(0.00, 0.03)
**Office blood pressure**				
Brachial SBP (mmHg)	135 ± 8	139 ± 11	−5	(−8, 1)
Brachial DBP (mmHg)	81 ± 7	85 ± 5	−4	(−6, −3)**
Brachial PP (mmHg)	54 ± 7	53 ± 8	1	(−3, 5)
MAP (mmHg)	103 ± 7	107 ± 8	−5	(−7, −2)**
Central SBP (mmHg)	118 ± 8	123 ± 11	−4	(−9, −1)*
Central DBP (mmHg)	95 ± 7	99 ± 6	−1	(−6, −2)**
Central PP (mmHg)	23 ± 4	24 ± 6	−1	(−3, 1)
HR (beats/min)	57 ± 7	58 ± 8	0	(−4, 2)
**Ambulatory blood pressure**				
24‐h SBP (mmHg)	126 ± 8	127 ± 9	−2	(−5, 1)
24‐h DBP (mmHg)	81 ± 5	82 ± 5	0	(−4, 1)
24‐h PP (mmHg)	46 ± 6	46 ± 7	−1	(−1, 2)
24‐h HR (beats/min)	69 ± 9	71 ± 11	0	(−4, 1)
**CGM outcomes**				
CGM_AUC_ (mmol/L*h)^a^	496 ± 103	477 ± 92	0	(−7, 44)
CGM_iAUC_ (mmol/L*h)^a^	75 ± 37	75 ± 24	0	(−20, 22)

*Note*. Values are means ± SD. Effect size is reported as the difference between the intervention and control period with a 95% confidence interval (95% CI; analysis of variance with a period as a covariate): *p < 0.05, **p < 0.01.

Abbreviations: AVR, arteriolar‐to‐venular ratio; CAIxHR75, central augmentation index adjusted for heart rate; CAR, carotid artery reactivity; CGM, continuous glucose monitor; CRAE, central retinal arteriolar equivalent; CRVE, central retinal venular equivalent; DBP, diastolic blood pressure; FMD, brachial artery flow‐mediated vasodilation; HR, heart rate; (i)AUC, (incremental) area under the curve; MAP, mean arterial pressure; net iAUC, net incremental area under the curve; PP, Pulse Pressure; PWV_c–f_, carotid‐to‐femoral pulse wave velocity; SBP, systolic blood pressure; Ye, Young's modulus of elasticity.

^a^ *n* = 14.

^b^ *n* = 16.

#### 3.2.2 Peripheral vasculature

FMD improved after the intervention as compared with the control period by 2.99 pp (95% CI: 0.58, 5.41 pp; *p* = 0.019), while the brachial artery diameter measured during the baseline period of the FMD decreased (Δ −0.34 mm; 95% CI: −0.17, −0.45 mm; *p* = 0.001) (Table 1; Figure 1c). The allometrically scaled FMD was not significantly affected (Δ 2.21 pp; 95% CI: −1.69, 6.18 pp; *p* = 0.241). An inverse correlation was observed between changes in FMD and CAR responses (*r* = −0.583, *p* = 0.029), while no significant correlation was observed between exercise‐induced changes in FMD and stiffness index β_0_ responses (*r* = 0.330, *p* = 0.249).

#### 3.2.3 Retinal microvasculature

The CRAE significantly increased by 4 μm (95% CI: 0, 7 μm; *p* = 0.041), while the CRVE did not change (Δ 1 μm; 95% CI: −1, 3 μm; *p* = 0.153). The AVR did not change (Δ 0.02; 95% CI: 0.00, 0.03; *p* = 0.076). The change in CRAE was inversely correlated with the change in brachial DBP (*r* = −0.53, *p* = 0.028) and nearly with central DBP (*r* = −0.477, *p* = 0.061). The correlation with changes in other blood pressure outcomes was not significant (MAP: *r* = −0.411, *p* = 0.101; central SBP: *r* = −0.342, *p* = 0.178).

#### 3.2.4 Cardiometabolic risk markers

**FIGURE 2:**
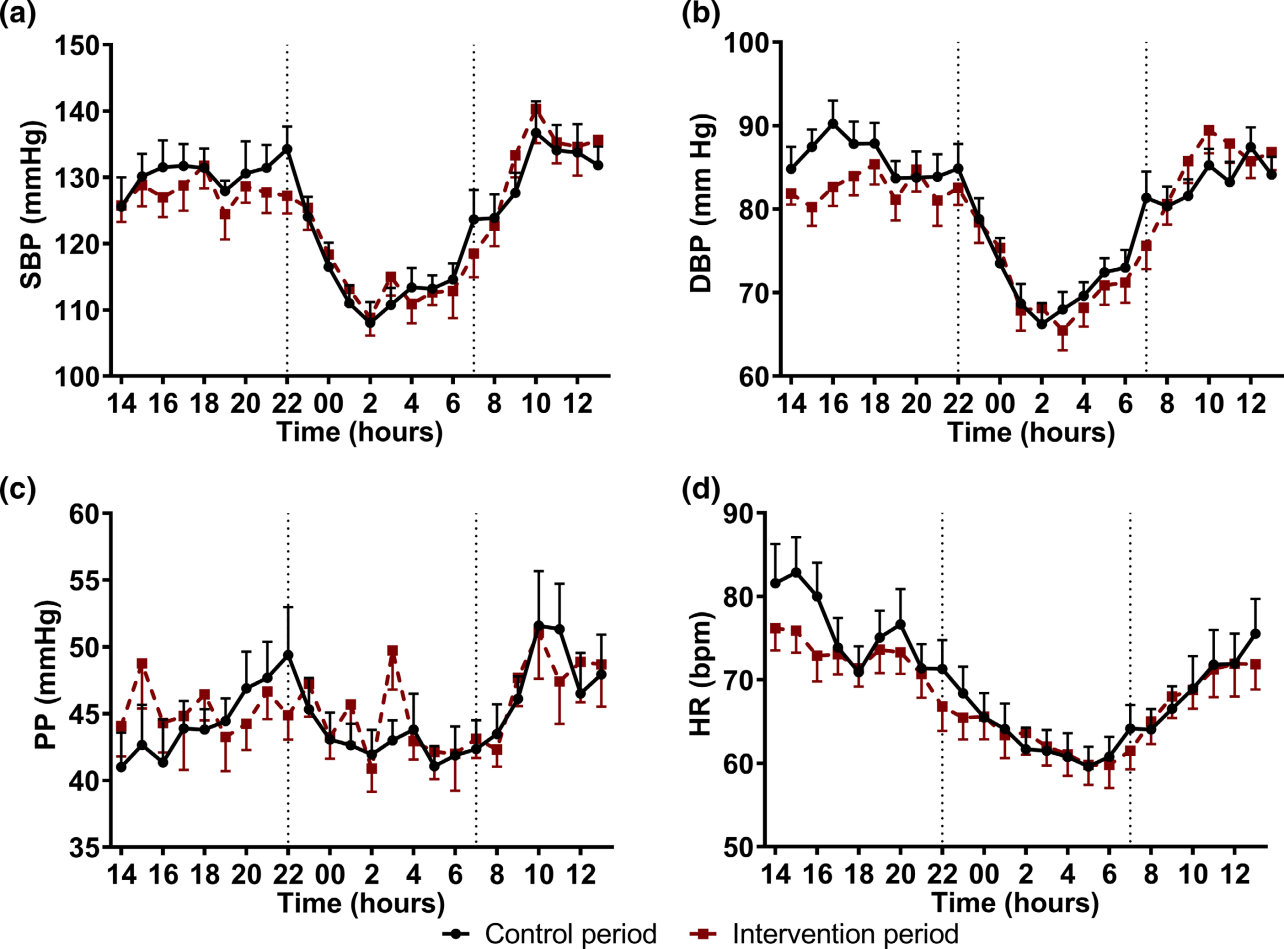
Mean 24‐h (±SEM) ambulatory blood pressure levels measured at the end of the exercise and control period in a randomized cross‐over study with sedentary overweight and obese older men (*n* = 17). Mean (a) systolic blood pressure (SBP), (b) diastolic blood pressure (DBP), (c) pulse pressure (PP), and (d) heart rate (HR) following the control and intervention period.

Office brachial DBP decreased by 5 mmHg (95% CI: −6, −3 mmHg; *p* = 0.002) after the intervention as compared with the control period. Central DBP also decreased by 5 mmHg (95% CI: −6, −2 mmHg; *p* = 0.001). Brachial SBP did not change (Δ 3 mmHg; 95% CI: −8, 1 mmHg; *p* = 0.096), while central SBP significantly decreased by 5 mmHg (95% CI: 1, 9 mmHg; *p* = 0.015). Office (Δ 1 mmHg; 95% CI: −3, 5 mmHg; *p* = 0.627) and central (Δ −1 mmHg; 95% CI: −3, 1 mmHg; *p* = 0.340) PP did not change. Additionally, MAP decreased by 4 mmHg (95% CI: −7, −2 mmHg; *p* = 0.005). Heart rate was comparable after both periods (Δ −1 beats/min; 95% CI: −4, 2 beats/min; *p* = 0.473). There were no significant effects on mean 24‐h (24 h), mean daytime, and mean night‐time ABP levels. Additionally, SDs and nocturnal dipping in SBP and DBP did not differ between both periods (Table 1; Figure 2 and Table S2).

There was a significant time × treatment effect for HDL‐cholesterol concentrations (*p* = 0.035), which increased by 0.07 mmol/L (95% CI: 0.01, 0.12 mmol/L; *p* = 0.015) in the intervention group as compared with the control group at week 4, while the difference in changes was comparable at week 8 (Δ 0.01 mmol/L; 95% CI: −0.04, 0.07; *p* = 0.599). TCH, LDL‐cholesterol, TAG, and hsCRP concentrations did not change (Table 2). No differences were observed at baseline (Table 2; TCH: *p* = 0.050, TAG: *p* = 0.772, HDL: *p* = 0.283, LDL: *p* = 0.065, CRP: *p* = 0.525). Finally, the Framingham Risk score did not differ (intervention period: 14.1 ± 2.3%, control period: 13.4 ± 1.9%; Δ 0.7%, 95% CI: −1.4, 0.2%; *p* = 0.110).

**TABLE 2:** Metabolic measurements from a randomized, controlled cross‐over study with sedentary obese older men (*n* = 17)

	Intervention period	Control period	Mean difference		*p*‐value	
	BL	BL	Δ WK4	Δ FU	Time × treatment	Treatment
TCH (mmol/L)	5.77 ± 1.06	5.55 ± 1.21	0.01 ± 0.49	−0.15 ± 0.92	0.648	0.547
TAG (mmol/L)	1.35 ± 0.47	1.36 ± 0.52	0.19 ± 0.57	0.09 ± 0.57	0.411	0.497
HDL (mmol/L)	1.33 ± 0.19	1.30 ± 0.16	−0.07 ± 0.12*	0.01 ± 0.13	0.035	N/A
LDL (mmol/L)	3.82 ± 0.97	3.64 ± 1.07	−0.02 ± 0.58	−0.21 ± 0.73	0.430	0.366
CRP (mg/L)^a^	3.35 ± 5.03	2.22 ± 2.30	−1.40 ± 3.54	−1.88 ± 4.71	0.332	0.099

*Note*. Values are means ± SD. Time × treatment and treatment effect (linear mixed model): *p < 0.05.

Abbreviations: BL, baseline; CRP, C‐reactive protein; FU, follow‐up; HDL, high‐density lipoprotein; LDL, low‐density lipoprotein; TAG, triacylglycerol; TCH, total cholesterol; WK4, week 4.

^a^ Statistics were performed after log‐transformation due to non‐normal distribution.

### 3.3 Continuous glucose monitoring and physical activity

The AUC (Δ 18 mmol/L*h; 95% CI: −7, 44 mmol/L*h; *p* = 0.139) and iAUC (Δ 0 mmol/L*h; 95% CI: −0.20, 0.22 mmol/L*h; *p* = 0.921) for continuous glucose concentrations over a 96‐h period did not significantly change (Table 1; Figure S3). No differences were observed between the intervention and control periods in physical activity score (Δ 0.01 MET/s; 95% CI: −0.02, 0.04 MET/s; *p* = 0.436) and sedentary time (Δ −24 s; 95% CI: −67, 18 s; *p* = 0.231).

## 4 DISCUSSION

In this randomized, controlled cross‐over trial with sedentary overweight and obese older men, aerobic exercise training improved endothelial function, as shown by the changes in FMD. The CAR response, which is related to effects on endothelial function and autonomic control of vascular tone, also improved. In contrast, local vascular stiffness of the carotid artery increased. Regional aortic vascular stiffness as measured with PWV_c–f_ did not change. Finally, retinal arteriolar diameters increased, and office blood pressure decreased. No effects were found on ABP, serum lipids, and 24‐h glucose concentrations. Participants did not change their habitual diet or regular daily physical activity pattern, underlining that the effects observed were due to the aerobic exercise training program. Moreover, exercise training had favorable effects on markers of the vasculature without concomitant alterations in the more conventional cardiometabolic risk markers.

Aerobic exercise training significantly improved the CAR response to a cold pressor test. In fact, the maximal vasodilation of the carotid artery increased to 4%, a value comparable to that of younger individuals aged 24 ± 3 years (van Mil et al., 2017). The increase in CAR response may decrease cardiovascular risk and the risk for future cardiovascular events (van Mil et al., 2017; Van Mil et al., 2019). Catecholamines (e.g., norepinephrine) released during the cold pressor test can increase vasodilation via endothelium‐dependent effects, but might at the same time cause vasoconstriction of smooth muscle cells via the sympathetic nervous system (Buckley et al., 2019). Thus, we can speculate that the balance between these two processes was beneficially affected after exercise training. The observed improvement in FMD, which is associated with a decreased CVD risk of 24% (Ras et al., 2013), is in line with the results of a meta‐analysis when a comparable training protocol was used (Early et al., 2017). Changes were not significant when accounting for the reduction in baseline diameter following exercise using allometric scaling. Importantly, ratio scaled FMD is associated with CVD risk (Ras et al., 2013), while the prognostic value is reduced when allometric scaling is applied (McLay et al., 2018). Moreover, a smaller baseline brachial artery diameter is also independently predictive of reduced cardiovascular risk (Maruhashi et al., 2018), underlining the clinical relevance of our findings. Taken together, observed changes in both major elastic conduit (CAR) and peripheral muscular arteries (FMD) support the evidence for beneficial effects of exercise training on vascular function of central and peripheral arteries, and thereby CVD risk in overweight and obese men. However, exercise‐induced changes in CAR and FMD responses were inversely correlated, providing evidence that different underlying mechanisms are involved.

Surprisingly, we observed that local carotid arterial stiffness increased. Although underlying mechanisms are unclear, it was hypothesized that repetitive elevations in blood pressure during training sessions, as well as adaptations in vascular tone, could have contributed to this observation (Tanaka, 2019). The observed changes in central blood pressure levels in our study may indeed impact arterial compliance. In contrast, central arterial stiffness was lower in participants who are more physically active (Seals et al., 2008; Tanaka et al., 2000). Also, daily walking for 14 weeks decreased the carotid arterial stiffness in normal‐weight older men (Tanaka et al., 2000). Further research on the effects of training on local artery stiffness is warranted.

PWV_c–f_, which is a marker for regional arterial stiffness, did not change. In contrast, a meta‐analysis of 20 studies showed an improved aortic PWV, which could not be explained by changes in blood pressure and was not related to exercise intensity (Ashor et al., 2014). However, this apparent discrepancy may be explained by our study duration of 8 weeks, as subgroup analyses revealed that improvements in PWV_c–f_ were only observed when the training program exceeded 10 weeks (Ashor et al., 2014). However, another study with an 8‐week intervention period found beneficial effects on PWV_c–f_ in healthy adults (Slivovskaja et al., 2018). Training sessions were, however, 10 min longer. This suggests that a longer time period or longer exercise training sessions may be needed to reduce stiffness in large central conduit arteries in healthy older men. In individuals with the metabolic syndrome, however, beneficial effects on PWV_c–f_ were observed after similar exercise training duration (Donley et al., 2014; Slivovskaja et al., 2018). Thus, a minimum amount of accumulated time seems to be needed to improve regional PWV_c–f_ in healthy volunteers. The positive effects on brachial‐ankle PWV that were observed (Ashor et al., 2014), may relate to increases in shear stress during aerobic exercise training, which may in particular affect nitric oxide‐producing muscular arteries (Ashor et al., 2014). Although stiffness index β_0_ and PWV both reflect arterial stiffness, these two parameters do not correlate (Tanaka, 2017). The lack of association may be due to differences in underlying mechanisms. The PWV is based on a propagation model involving regional measurements that track movements of pulse waves from one location to another along the arterial tree, whereas the stiffness index β_0_ represents a pulsation model consisting of local assessment using ultrasound. As compared with the PWV, stiffness index β_0_ is also less dependent on blood pressure (Lim et al., 2015), which was decreased in this study. Moreover, arterial stiffness also varies depending on the location of the measurements (van Popele et al., 2001).

Retinal microvascular calibers were beneficially affected, as shown by an increased CRAE, although CRVE did not change. These results agree with those of other studies (Hanssen et al., 2011; Köchli et al., 2018), while an inverse correlation between changes in CRAE and DBP could also be established. Interestingly, wider retinal arterioles have previously been related to a decreased risk to develop hypertension (Ding et al., 2014).

Office blood pressure decreased after aerobic exercise training, which was consistent with previous studies that focused on office blood pressure and is related to a reduction in CVD risk (Cornelissen & Fagard, 2005). It is suggested that a reduction in vascular resistance via the sympathetic nervous system and the renin‐angiotensin system are involved. In contrast, ABP was not affected. A meta‐analysis of 15 aerobic exercise training studies of at least 4 weeks observed a significant, albeit modest, decrease of 3 mmHg in daytime SBP and DBP (Cornelissen et al., 2013). It is possible that the longer median study duration of 15 weeks (range: 6–52 weeks) and the reduction in body weight in one‐third of the studies included in that meta‐analysis may explain this discrepancy (Cornelissen et al., 2013). It can also be speculated that only office BP is reduced after exercise training during a resting period and not ABP during daily activities, due to increased sympathetic activity (Van Hoof et al., 1989).

We have already reported improvements in post‐load glucose concentration during an oral glucose tolerance test (Kleinloog et al., 2019). However, fasting glucose and insulin concentrations and the homeostatic model assessment index as a measure of insulin resistance did not change (Kleinloog et al., 2019). We now found that CGM and the serum lipid profile did not change following exercise training. In our study, body weight remained stable, while improvements in the serum lipid profile in other studies often coincided with weight reduction (Bateman et al., 2011; Katzmarzyk et al., 2003; Malin et al., 2012; Yassine et al., 2009). Beneficial effects in these studies may, therefore, not be attributed to the exercise intervention alone. In addition, our study population may have been too healthy to improve metabolic risk markers. Indeed, Couillard et al. (2001) only observed the beneficial effects of an exercise intervention on these serum lipids in participants with metabolic disorders.

This randomized, controlled, cross‐over trial had a wash‐out period of 12 weeks. Although VO_2_peak returned to baseline, we cannot exclude that some outcome parameters had not yet returned to baseline after the wash‐out period. The decrease in brachial baseline diameters may have increased shear stress resulting in an increased FMD. Unfortunately, we did not determine flow stimuli and reactive hyperemia data can thus not be reported. Estimates of the carotid diameter were lower during the baseline period of the CAR compared with assessments during five‐to‐six heartbeats, which may be due to the differences in the software used. Multiple, possibly interrelated, markers for vascular function were affected, while we also observed effects on office blood pressure, which makes it impossible to estimate the overall effect on CVD risk reduction. Also, we might have been underpowered to detect differences in some of the described outcome parameters. This trial was performed only on overweight and obese men with an age between 60 and 70 years to reduce gender differences as an extra source of variability in particular for the primary outcome parameter of this study (CBF), which has been published before (Kleinloog et al., 2019).

This trial provides evidence that aerobic exercise training in sedentary overweight and obese older men improves not only FMD but also CAR responses and retinal arteriolar width. These effects may be important mechanisms by which aerobic exercise training reduces age‐related health problems, such as CVD and cognitive decline. The results of exercise training on local carotid stiffness warrant further study.

## AUTHOR CONTRIBUTIONS

The authors' contributed as follows; JK: designed and conducted the study, performed the statistical analyses, interpreted the data, and wrote the manuscript, RM: designed the study, interpreted the data, had overall responsibility for the study, and wrote the manuscript, JR: set up vascular function measurements, interpreted the data, and reviewed the manuscript, DT: set up the CAR measurement, interpreted the data, and reviewed the manuscript, MH: designed the exercise protocol, and reviewed the manuscript, and PJ: designed the study, interpreted the data, had overall responsibility for the study, and wrote the manuscript.

## CONFLICT OF INTEREST

The project is organized by and executed under the auspices of TiFN, a public‐private partnership on precompetitive research in food and nutrition. Funding was obtained from the Netherlands Organization for Scientific Research. The authors have declared that no competing interests exist. The datasets analyzed during the current study are available from the last author on reasonable request.

## Supporting information


**Appendix S1** Supporting information
