# Supplementary material for: Aerobic exercise training improves not only brachial artery flow‐mediated vasodilatation but also carotid artery reactivity: A randomized controlled, cross‐over trial in older men
Source: Physiol Rep. 2022 Aug 27;10(16):e15395. doi: 10.14814/phy2.15395 (PMC9419153; doi:10.14814/phy2.15395)
Supplement: Supplementary file 1 — Appendix S1 Supporting information [file PHY2-10-e15395-s001.docx]

**Supplementary Information**

**

**

**Supplementary Figure 1.** Schematic overview of study design. Timeline is displayed as weeks.

**

**

**Supplementary Figure 2.** Consort flow diagram. Diagram of the progress through the phases of this randomized, controlled cross-over trial with two periods. FMD: flow-mediated vasodilation, CAR: carotid artery reactivity, CGM: continuous glucose monitor.

**
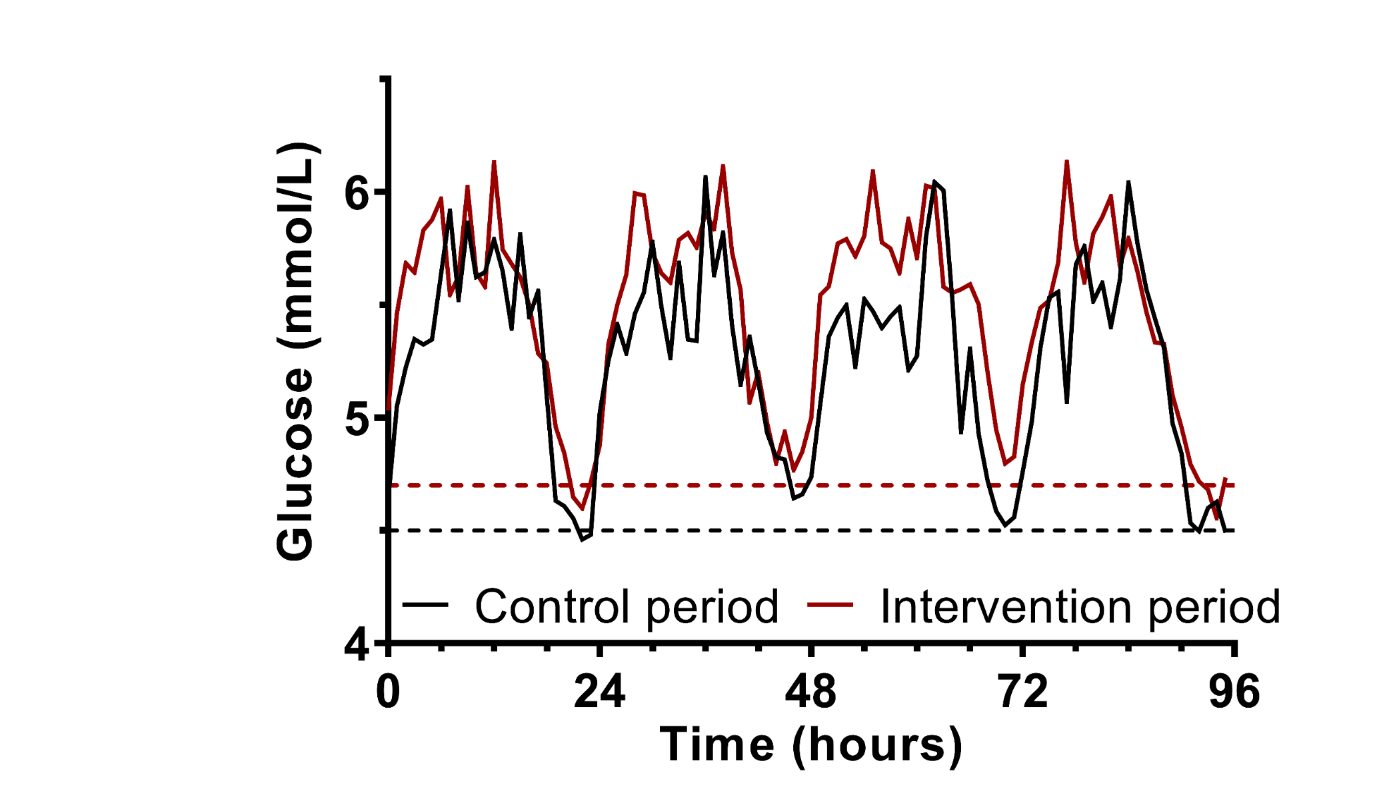
Supplementary Figure 3.** Mean 96-hour continuous glucose measurements obtained from a randomized, controlled cross-over study with sedentary older men (n = 16) following the intervention and control period. The horizontal dashed lines (black = control period, red = intervention period) represent the average fasting glucose concentration that was calculated during the nights from 03:00 to 04:00 hour.

| **Supplementary Table 1.** Average energy and nutrient intakes after an exercise period or no-exercise control period from a randomized, controlled cross-over study with sedentary older men (n = 17). | | | | | | | | |
| --- | --- | --- | --- | --- | --- | --- | --- | --- |
|  | **Intervention period** | | | **Control period** | | | **Mean difference**  **(95% CI)** | |
| Energy (KJ/day) | 9320 | ± | 1749 | 9770 | ± | 2666 | -640 | (-1,648, 369) |
| Energy (Kcal/day) | 2224 | ± | 418 | 2332 | ± | 638 | -152 | (-393, 88) |
| Carbohydrate (En%) | 36.7 | ± | 7.1 | 36.7 | ± | 7.8 | -0.2 | (-3.5, 3.2) |
| Protein (En%) | 16.8 | ± | 3.8 | 17.6 | ± | 5.7 | -0.7 | (-2.7, 1.3) |
| Total fat (En%) | 39.6 | ± | 5.2 | 38.9 | ± | 5.3 | 0.6 | (-1.7, 2.8) |
| Saturated FA (En%) | 13.5 | ± | 2.2 | 13.6 | ± | 2.0 | -0.1 | (-0.8, 0.5) |
| Monounsaturated FA (En%) | 15.0 | ± | 4.9 | 14.2 | ± | 4.4 | 0.8 | (-1.0, 2.5) |
| Polyunsaturated FA (En%) | 7.5 | ± | 1.5 | 7.4 | ± | 1.7 | 0.0 | (-0.5, 0.6) |
| Alcohol (En%) | 4.7 | ± | 4.8 | 4.7 | ± | 4.8 | 0.1 | (-0.6, 0.8) |
| Dietary fiber (g/day) | 24.5 | ± | 7.8 | 22.0 | ± | 8.5 | 1.8 | (-1.2, 4.8) |
| Values are means ± SD. The mean difference is corrected for period (Repeated analysis of variance with period as fixed factor). 95% CI: 95% confidence interval; En%: Energy percentage; FA: fatty acids. | | | | | | | | |

| **Supplementary Table 3.** Ambulatory blood pressure measurements after an exercise period or no-exercise control period from a randomized, controlled cross-over study with sedentary older men (n = 17). | | | | | | | | |
| --- | --- | --- | --- | --- | --- | --- | --- | --- |
|  | **Intervention period** | | | **Control period** | | | **Mean difference (95% CI)** | |
| Daytime SBP (mmHg) | 129 | ± | 8 | 129 | ± | 10 | 0 | (-5, 2) |
| Daytime DBP (mmHg) | 83 | ± | 5 | 84 | ± | 6 | -2 | (-4, 0) |
| Daytime PP (mmHg) | 46 | ± | 6 | 46 | ± | 7 | 1 | (-2, 2) |
| Daytime HR (beats/min) | 71 | ± | 9 | 73 | ± | 12 | -2 | (-5, 2) |
| Nighttime SBP (mmHg) | 117 | ± | 8 | 115 | ± | 9 | 1 | (-5, 3) |
| Nighttime DBP (mmHg) | 73 | ± | 6 | 73 | ± | 6 | 0 | (-4, 3) |
| Nighttime PP (mmHg) | 44 | ± | 5 | 43 | ± | 7 | 1 | (-2, 2) |
| Nighttime HR (beats/min) | 63 | ± | 10 | 63 | ± | 9 | 0 | (-3, 2) |
| SD 24-hour SBP (mmHg) | 15 | ± | 3 | 16 | ± | 4 | -1 | (-4, 1) |
| SD 24-hour DBP (mmHg) | 11 | ± | 2 | 11 | ± | 2 | 0 | (-2, 1) |
| SD 24-hour PP (mmHg) | 11 | ± | 4 | 12 | ± | 4 | -1 | (-2, 1) |
| SD 24-hour HR (beats/min) | 9 | ± | 3 | 11 | ± | 3 | -1 | (-3, 1) |
| SD Daytime SBP (mmHg) | 15 | ± | 4 | 15 | ± | 4 | 0 | (-4, 2) |
| SD Daytime DBP (mmHg) | 10 | ± | 2 | 10 | ± | 2 | 0 | (-1, 1) |
| SD Daytime PP (mmHg) | 12 | ± | 4 | 13 | ± | 4 | -1 | (-3, 2) |
| SD Daytime HR (beats/min) | 10 | ± | 3 | 11 | ± | 4 | -1 | (-3, 2) |
| SD Nighttime SBP (mmHg) | 12 | ± | 3 | 12 | ± | 4 | 0 | (-4, 1) |
| SD Nighttime DBP (mmHg) | 10 | ± | 2 | 10 | ± | 3 | 0 | (-2, 1) |
| SD Nighttime PP (mmHg) | 8 | ± | 4 | 7 | ± | 3 | 1 | (-1, 3) |
| SD Nighttime HR (beats/min) | 5 | ± | 2 | 6 | ± | 3 | -1 | (-2, 0) |
| Dipping SBP (%) | 10 | ± | 4 | 10 | ± | 6 | 0 | (-4, 3) |
| Dipping DBP (%) | 13 | ± | 5 | 13 | ± | 6 | 0 | (-5, 2) |
| Values are means ± SD. The mean difference is corrected for period (Repeated analysis of variance with period as fixed factor). 95% CI: 95% confidence interval (analysis of variance with period as covariate). DBP: diastolic blood pressure; HR: heart rate; PP: pulse pressure; SD: standard deviation of individual 24-hour daytime or nighttime values (within-subject variability); SBP: systolic blood pressure. | | | | | | | | |
